# Label-free estimation of regulatory T cell activation markers using Raman spectroscopy with machine learning

**DOI:** 10.1038/s41598-025-16002-8

**Published:** 2025-11-04

**Authors:** Aria Azari-Pour, Ali Chamkalani, Shreyas Rangan, Katherine N. MacDonald, Miles Huynh, Megan K. Levings, H. Georg Schulze, James M. Piret, Bhushan Gopaluni

**Affiliations:** 1 Center for Misfolding Diseases, Yusuf Hamied Department of Chemistry, University of Cambridge, Lensfield Road, Cambridge, CB2 1EW UK; 2 Michael Smith Laboratories, University of British Columbia, 2185 E Mall, Vancouver, BC V6T 1Z4 Canada; 3 Department of Biochemistry and Molecular Biology, Faculty of Medicine, University of British Columbia, Life Sciences Centre, 2350 Health Sciences Mall, Vancouver, BC V6T 2A1 Canada; 4 Department of Chemical and Biological Engineering, Faculty of Applied Science, University of British Columbia, 2360 East Mall, Vancouver, BC V6T 1Z3 Canada; 5 School of Biomedical Engineering, University of British Columbia, 2222 Health Sciences Mall, Vancouver, BC V6T 2B9 Canada; 6 BC Children’s Hospital Research Institute, 938 W 28th Ave, Vancouver, BC V5Z 4H4 Canada; 7 Department of Surgery, Faculty of Medicine, University of British Columbia, 2775 Laurel Street, Vancouver, BC V5Z 1M9 Canada; 8 5823 Schooner Way, Pender Island, BC V0N 2M0 Canada

**Keywords:** T cells, Activation markers, Raman spectroscopy, Machine learning, Estimation

## Abstract

Regulatory T cells are a class of T lymphocytes which respond to activation signals by expanding their cell numbers, and whose culturing and expansion are of significant clinical interest. Cellular activation states are used to inform process control decisions such as restimulation and can be probed with experimental measurements of cell surface markers. However, these measurements are expensive, time-consuming, and invasive, and an urgent need exists for devising a non-invasive method for activation state monitoring that could be deployed on-line. Raman spectroscopy is a label-free and information-rich optical method that, when coupled to data analytical methods, can ameliorate these experimental issues. In this work, we quantitatively estimated experimental measurements of regulatory T cell activation markers with high accuracy. We simulated a clinical manufacturing setting by building an ${L}_{1}$-regularized least-squares model with spectroscopic data from six regulatory T cell donors. Then, we validated the constructed model by accurately estimating different experimental measurements of biomarker values from two external donors, unseen by the model. We have devised a robust program to effectively estimate the activation state of regulatory T cells. We anticipate our method to be used with on-line Raman probes integrated into cell manufacturing devices for label-free monitoring of these processes.

**Supplementary Information:**

The online version contains supplementary material available at 10.1038/s41598-025-16002-8.

## Introduction

Immune cell therapies have emerged as a viable candidate for the treatment of human diseases^1,2^. The culturing and expansion of cells for immune cellular therapies is a critical process control parameter which impacts the efficacy of these therapies^3–6^. In clinical settings during culturing and expansion, cells will typically be activated through stimulation with specific antigen-presenting cells showing a specific antigen. The subsequent timeline of maintaining these cells requires dynamic time-dependent information regarding the intracellular growth state of the expanding culture^7,8^. Typically following the initial activation, at some point during the expansion the intracellular growth rate slows and the cells must be restimulated^9,10^. The standard method to understand the intracellular dynamics is with experimental measurements such as flow cytometry, which are costly, time-consuming, and require advanced equipment^11–13^. There is a pressing need to devise alternative methodologies to probe the activation states of immune cells during clinical culturing and expansion^14,15^. In this work, we use simple regression methods with measured Raman spectroscopy data to quantitatively estimate experimental measurements of known regulatory T (Treg) cell activation markers.

Tregs are a subpopulation of T lymphocytes that maintain immune homeostasis and prevent autoimmunity^16^. Tregs comprise 5-10% of the CD4+ T cell population and can be characterized by their expression of CD4, CD25, and FOXP3 transcription factors^17^. These specialized immune cells modulate immune responses to suppress immune activation^18–20^. Cell therapies with Tregs are currently undergoing clinical trials to treat autoimmune disorders, transplant rejection, and graft-versus-host disease^21^. Tregs have also been transduced to express a Chimeric Antigen Receptor (CAR) for CAR-Treg therapy^22^, building on the success of CAR T cell therapy cancer treatment^23^.

The latency-associated peptide (LAP) is a Treg biomarker which is part of the latent form of transforming growth factor-β1 (TGF-β1) and in turn controls T cell development, homeostasis, and function^24^. TGF-β1 is a suppressive cytokine expressed by Tregs that is secreted upon proteolytic cleavage of the latent, LAP-complexed form^25^. Glycoprotein A repetitions predominant (GARP), another Treg biomarker, is a transmembrane protein containing leucine-rich repeats that can bind and present latent TGF-β1 on the cell surface^26^. Both LAP and GARP are highly expressed on the surface of activated Tregs^26^, and we aim to estimate the levels of flow cytometry measurements of these two biomarkers in this study. When expanding the number of Tregs in culture, decreases in the levels of these markers provide an indication of slowing Treg growth, such that their levels can be used to guide when to restimulate the Tregs for greater expansion in their numbers^27^. If cells are restimulated too early, this is known to cause activation-induced cell death^28^. Thus, it would be useful if the levels of these Treg activation biomarkers could be analyzed by an on-line method, to not depend on frequent sampling that require off-line flow cytometry analysis.

Raman spectroscopy is an experimental method that can generate a large amount of information about a biological system. Spectroscopic methods have the advantage that they can be implemented non-invasively, non-destructively, and cost-effectively compared to other means of chemometric analysis. Raman spectroscopy is based on Raman scattering, a phenomenon of light-matter interaction in which light is inelastically scattered, resulting in a different wavelength after scattering due to an exchange of energy^29^. Raman scattering was first predicted by Smekal^30^ and then observed experimentally by Raman and Krishnan^31^. A Raman spectrum is a plot of the scattering intensity as a function of the light frequency shift after Raman scattering, referred to as a Raman shift and measured in wavenumbers^29^. Talari et al.^32^ provide a review on biological assignments of Raman shifts.

The complexity and multidimensionality of biological Raman spectra require advanced regression techniques to decipher and extract meaningful information. Data-driven machine learning (ML) modelling approaches excel at recognizing intricate patterns and relationships within high-dimensional datasets^33–35^. ML techniques can efficiently process large amounts of data and handle non-linearities. By training ML models on large-scale Raman spectral data, these models can learn to discern subtle spectral features, capture non-linear dependencies, and uncover hidden structures that may elude traditional analytical approaches. Leveraging these methods allows for more accurate classification, prediction of unknown spectra, and identification of structural changes or interactions. The utilization of ML methods for analyzing Raman spectral data has been shown to be effective^36–38^. In oncology, substantial research has been carried out on the ability to classify cancers and other diseases using Raman spectroscopic data^36^.

The main question of interest in this work was formulated as follows. Is it possible to use the intensities in the Raman spectra of Tregs to estimate the levels of LAP and GARP in Tregs to permit substituting measurements of these Treg biomarkers with measurements of Raman spectra? Such a capability could complement or even circumvent laboratory methods to perform real-time analysis, enabling rapid decision-making in applications such as quality control, process optimization, and characterization. In this work, a preliminary analysis was performed using Multisource Correlation Analysis^39^. The results of this linear method led to the investigation of the problem more formally using five linear supervised ML methods. Three methods were variations on the least-squares algorithm: Ordinary Least-Squares (OLS), Least Absolute Shrinkage and Selection Operator (Lasso), and Ridge Regression; the fourth was Partial Least-Squares (PLS) Regression, a combination of Principal Component Analysis (PCA) and OLS; and the final model was Linear Support Vector Regression (LSVR).

In the next section, we formulate the mathematical statement of the problem of interest in this paper. The input and output data for our workflow will be formally described in the context of the theory of the different least-squares models, and we refer readers to the literature on both PLS^40^ and LSVR^41^.

### Modelling and theoretical aspects

A Raman spectrum is denoted as ${\left({X}_{j}\right)}_{j=1}^{m}\in {\mathbb{R}}^{m}$, and is a vector in *m*-dimensional Euclidean space with components given by the experimentally measured spectral intensities $\left\{{X}_{j}:j=1,\dots ,m\right\}$ at each of the Raman shifts $j=1,\dots ,m$. There is a fixed number of $m$ Raman shifts, or features, that are measured in each Raman spectrum, which in this paper is always $m=963$. A collection of *n* Raman spectra, where *n* denotes the number of statistical samples, is denoted with boldface as $\mathbf{X}=\left({X}_{ij}\right)\in {\mathbb{R}}^{n\times m}$ and is called the regressor (or design) matrix with *n* rows and *m* columns. For each statistical sample $i=1,\dots ,n$, there is, at the same time point, a corresponding response variable, and all the response variables together represent the response vector, ${\mathbf{Y}=\left({Y}_{i}\right)}_{i=1}^{n}\in {\mathbb{R}}^{n}$. A full dataset is defined as $\left\{\mathbf{Y}, \mathbf{X}\right\}={\{{Y}_{i}, {X}_{i1}, \dots , {X}_{im}\}}_{i=1}^{n}$, i.e., the total number of input features and responses measured across all samples. Additionally, we have used eight Treg donors for data acquisition where the data obtained, i.e., the Raman spectra and biomarker response variables, were measured over six days. Therefore, the number of statistical samples will be $n\le 48$ for most purposes.

The problem of interest of this paper can now be stated mathematically. Suppose we have a full dataset $\left\{\mathbf{Y}, \mathbf{X}\right\}$, and a separate collection of *k* measured Raman spectra $\overline{\mathbf{X}}\in {\mathbb{R} }^{k\times m}$ for which we may have no measured response data for all or some of the samples. Then, given a set of parameters $\Theta$, we want to build a parametric function $f:{\mathbb{R}}^{k\times m}\to {\mathbb{R}}^{k}$ which accurately estimates the response variables $\overline{\mathbf{Y} }$ corresponding to $\overline{\mathbf{X} }$, or in other words, what would have been measured had we performed an experimental measurement for those samples. The mathematical statement of the problem above does not specify the functional form of $f=f(\overline{\mathbf{X} };\Theta )$, however, we assume that the function $f$ is linear in its argument $\overline{\mathbf{X} }$. Let us introduce the parameters $\Theta =\left\{{\beta }_{1},\dots ,{\beta }_{m}, {\beta }_{0}\right\}=\left\{{\varvec{\upbeta}}, {\beta }_{0}\right\}$ and refer to ${\varvec{\upbeta}}={\left({\beta }_{1},\dots ,{\beta }_{m}\right)}^{\text{T}}\in {\mathbb{R}}^{m}$ as a weight vector, with ${\beta }_{0}\in {\mathbb{R}}$ a constant. Then a linear parametric model assumes that for the ${i}^{\text{th}}$ sample of some design matrix $\mathbf{X}$ the response has the form

1 $${{Y}}_{\text{i}}^{{^{\prime}}}={\beta }_{1}{{X}}_{{i}1}+\dots +{\beta }_{{m}}{{X}}_{{im}}+{\beta }_{0}$$

In vector form, the estimated response vector ${\mathbf{Y}}^{\mathbf{^{\prime}}}$ can therefore be written as

2 $${{\mathbf{Y}}}^{{^{\prime}}}={\mathbf{X}}{\varvec{\upbeta}}+{\beta }_{0}{1}_{{\boldsymbol{k}}}$$

where ${1}_{{\boldsymbol{k}}}$ is the *k*-dimensional vector with all entries equal to 1. The quantity ${\beta }_{0}$ is referred to as a bias term or offset in the estimation. Eq. (2) above is computed easily using matrix multiplication assuming that the optimal weights ${\varvec{\upbeta}}$ and offset ${\beta }_{0}$ are known. The mathematical problem has now shifted to finding parameters $\Theta =\left\{{\varvec{\upbeta}}, {\beta }_{0}\right\}$ which give the most accurate estimates. The use of least-squares regression to find optimal parameters $\Theta$ can be summarized as follows: we have a dataset $\left\{\mathbf{Y},\mathbf{X}\right\}$ and we want to use this dataset to generate the parameters $\Theta$ based on the patterns in the data. Then, those parameters $\Theta$ can be used with $\mathbf{X}$ in Eq. (2) to obtain the estimate ${\mathbf{Y}}^{\mathbf{^{\prime}}}$. The process of finding optimal parameters $\Theta$ from the dataset $\left\{\mathbf{Y},\mathbf{X}\right\}$ is referred to as training the model $f$ and $\left\{\mathbf{Y},\mathbf{X}\right\}$ is referred to as the training set. The process of validating the trained model by using it to estimate responses from samples it was not trained with is referred to as testing the model $f$. In this work, we will refer to the process of exporting the trained model to estimate responses for donors that are completely novel to the model as validation or external donor validation.

### Least-squares model and regularization

The least-squares algorithm finds the parameters $\Theta$ which minimize the distance between the estimated responses given by $\Theta$ and the training responses. Let

3 $${\Vert {\mathbf{Q}}\Vert }_{{p}}:={\left(\sum_{{i}=1}^{{n}}{\left|{{Q}}_{{i}}\right|}^{{p}}\right)}^{1/{p}}$$

be the *L*_p_ norm of a vector $\mathbf{Q}=\left({Q}_{1},\dots ,{Q}_{n}\right)\in {\mathbb{R}}^{n}$. Now, introduce the least-squares loss function as

4 $$\mathcal{O}\left({\varvec{\upbeta}},{\beta }_{0}\right)={\left({\Vert {\mathbf{Y}}-{{\mathbf{Y}}}^{{^{\prime}}}\Vert }_{2}\right)}^{2}=\sum_{{i}=1}^{{n}}{\left({{Y}}_{{i}}-{{Y}}_{{i}}^{{^{\prime}}}\right)}^{2}=\sum_{{i}=1}^{{n}}{\left({{Y}}_{{i}}-\sum_{{j}=1}^{{m}}{{X}}_{{ij}}{\beta }_{{j}}-{\beta }_{0}\right)}^{2}$$

The more accurate the estimate $\mathbf{Y}{^{\prime}}$, the smaller $\mathcal{O}\left({\varvec{\upbeta}},{\beta }_{0}\right)$. Therefore, the problem has now shifted to minimizing $\mathcal{O}\left({\varvec{\upbeta}},{\beta }_{0}\right)$. The loss function depends only on the parameters ${\varvec{\upbeta}}, {\beta }_{0}$, as the training dataset $\left\{\mathbf{Y},\mathbf{X}\right\}$ is fixed and $\mathbf{Y}{^{\prime}}$ depends on $\mathbf{X},{\varvec{\upbeta}},\text{ and }{\beta }_{0}.$ For the OLS model, the optimal parameters, denoted by $\widehat{\Theta }=\left\{\widehat{{\varvec{\upbeta}}}, {\widehat{\beta }}_{0}\right\},$ can be found as the minimum of $\mathcal{O}\left({\varvec{\upbeta}}, {\beta }_{0}\right)$ with respect to the arguments ${\varvec{\upbeta}}, {\beta }_{0}$, formally written as

5 $$\widehat{\Theta }=\left\{\widehat{{\varvec{\upbeta}}}, {\widehat{\beta }}_{0}\right\}=\underset{{\varvec{\upbeta}}, {\beta }_{0}}{{\arg}\; {\min}}\mathcal{O}\left({\varvec{\upbeta}}, {\beta }_{0}\right)$$

There is an analytical solution to Eq. (5), however, this may not be practical. In this study, we have $m\gg n$, i.e., the number of features far exceeds the number of samples. Such a phenomenon often leads to overfitting, in which the model is trained with high accuracy for the training responses but performs with low accuracy for testing responses. In such a low-*n* type situation, it is often beneficial to use regularization, in which one amends the loss function in order to encourage the model to tend to solutions of ${\varvec{\upbeta}}, {\beta }_{0}$ in which many components of ${\varvec{\upbeta}}$ are zero.

The first regularized model we consider is Lasso, whose loss function has the form

6 $$\mathcal{L}\left({\varvec{\beta}}, {\beta }_{0};\lambda \right)=\mathcal{O}\left({\varvec{\upbeta}}, {\beta }_{0}\right)+\lambda {\Vert {\varvec{\upbeta}}\Vert }_{1}$$

and the parameters $\Theta$ are found according to Eq. (5) with Eq. (6) instead as the loss function. The value $\lambda$ is referred to as a hyperparameter and is chosen, or tuned, before training. The second regularized model we consider is Ridge, which has a loss function of the form

7 $$\mathcal{R}\left({\varvec{\upbeta}}, {\beta }_{0};\delta \right)=\mathcal{O}\left({\varvec{\upbeta}}, {\beta }_{0}\right)+\delta {\left({\Vert {\varvec{\upbeta}}\Vert }_{2}\right)}^{2}$$

and again, the parameters are found according to Eq. (5) with Eq. (7) instead as the loss function, and $\delta$ is a hyperparameter.

In this work, the density of a vector refers to the number of non-zero components and the sparsity of a vector refers to the number of components that are equal to zero, such that the sparsest vectors are $0$-dense. Therefore, a vector is $k$-dense if it contains exactly $k$ non-zero components and all other components are zero. We refer to feature $i$ being selected after training a model if the ${i}^{\text{th}}$ component of the weight vector is non-zero, ${\beta }_{i}\ne 0$. If $k$ features are selected, then the resulting vector ${\varvec{\upbeta}}$ will be $k$-dense and $\left(\text{dim}{\varvec{\upbeta}}-k\right)$-sparse. Lasso is well-known to enhance sparsity in vector-based feature selection within high-dimensional feature spaces^42^.

In order to quantify the contribution of a single feature to the estimation of a biomarker, we define the Biomarker Estimation Contribution (BEC) of feature $j=1,\dots ,m$ for sample $i=1,\dots ,n$ as the product ${\beta }_{j}{X}_{ij}$. Summing the BECs over all the features of the ${i}^{\text{th}}$ sample gives the estimate of the ${i}^{\text{th}}$ response minus the bias, ${Y}'_{i}-{\beta }_{0}=\sum_{j=1}^{m}{\beta }_{j}{X}_{ij}$. The weight ${\beta }_{j}$ for feature $j$ may be very high, but if the Raman spectral intensity, ${X}_{ij}$, of that feature for sample $i$ is very low, then the contribution to the biomarker estimation of feature $j$ for that sample is low. For a set of $k$ samples indexed by $\{1, \dots , k\}$, we define the mean BEC and standard deviation (SD) BEC of feature $j$ as

8 $$\text{Mean BEC}={\beta }_{j}{\langle {X}_{ij}\rangle }_{i}\equiv \frac{{\beta }_{j}}{k}\sum_{i=1}^{k}{X}_{ij}$$

9 $${\left(\text{SD BEC}\right)}^{2}\equiv \frac{1}{k-1}\sum_{i=1}^{k}{\left({{\beta }_{j}X}_{ij}-{{\beta }_{j}\langle {X}_{nj}\rangle }_{n}\right)}^{2}$$

where ${\langle {X}_{nj}\rangle }_{n}$ denotes the arithmetic mean over the index $n$.

## Results

### Treg expression and spectroscopic data

**Fig. 1:**
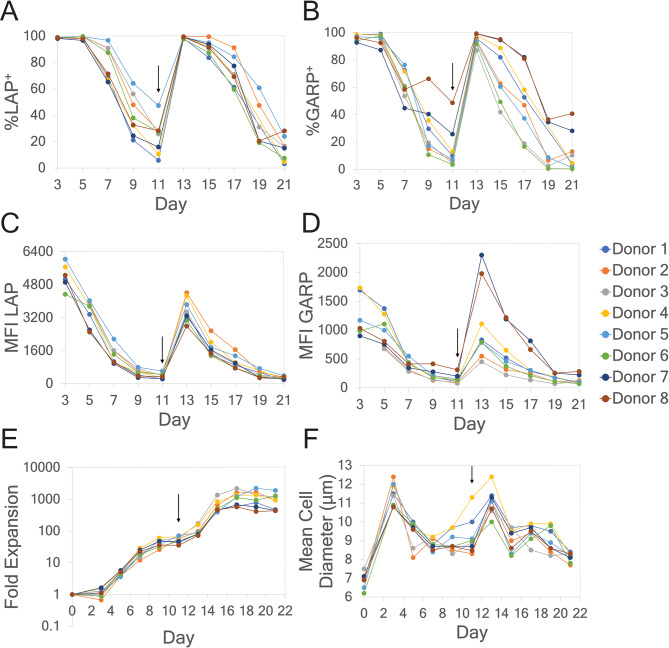
Measurements of Tregs biomarkers and properties for eight different Treg donors. The biomarkers shown are (**A**) %LAP^+^, (**B**) %GARP^+^, (**C**) MFI LAP, (**D**) MFI GARP, and the cellular properties shown are (**E**) fold expansion, and (**F**) mean cell diameter. Biomarkers **A**–**D** were measured using flow cytometry and both percent positive (%^+^) and mean fluorescence intensity (MFI) are shown. The full culturing timeline is shown, but only days 5-15 were used for chemometric analysis in this paper. Thus, day 0 measurements using flow cytometry are not shown. The culturing timeline was 0–21 days post-activation, with sampling on days 0, 3, 5, 7, …, 21. The restimulation on day 11 is indicated by the black arrow.

**Fig. 2:**
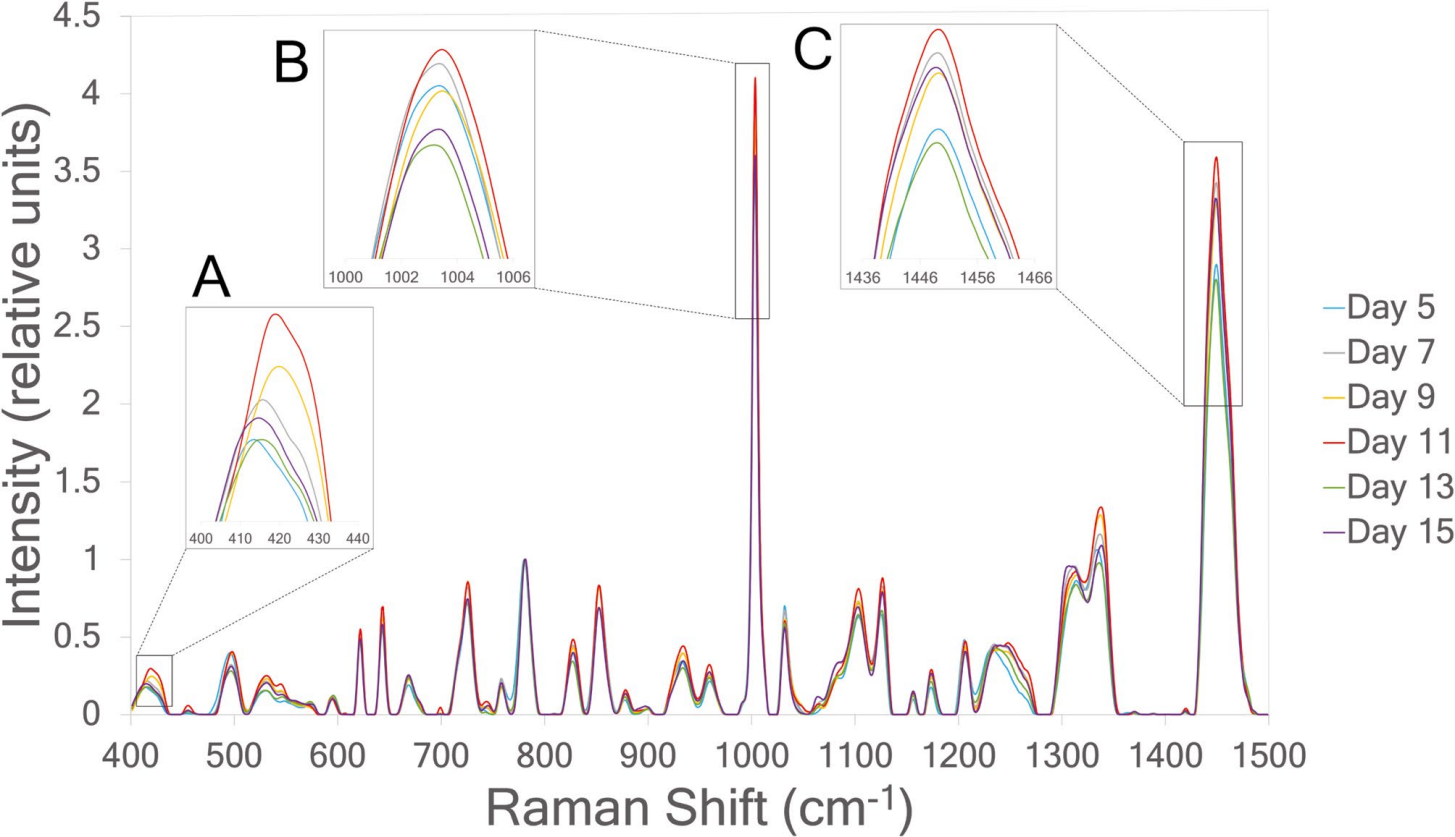
Mean Raman spectra of Treg Donor 5 on days 5–15 post-activation, with restimulation on day 11. The spectral intensities are normalized to nucleic acids, wherein for each Raman shift, the relative intensity on the vertical axis is the ratio of its mean spectral intensity to the intensity of the most intense peak within a neighborhood (defined as $\pm$ 2 wavenumbers on either side) of the 782 cm^-1^ peak, which is a nucleic acid band (DNA, RNA, nucleotide bases). The insets are zoomed in plots at different biologically important regions of the spectrum which vary along the culturing timeline: (**A**) a phosphatidylinositol band around 415 cm^-1^ and a cholesterol band around 418 cm^-1^; (**B**) a phenylalanine band around 1003 cm^-1^; and (**C**) a protein band around 1450 cm^-1^.

Tregs respond to activation signals by expanding their cell numbers for 7 to 14 days before return to a resting state. To obtain greater expansion, they must be restimulated or activated again. Figure 1A–D shows flow cytometric measurements of LAP and GARP with two different metrics, percent positive (%^+^) and mean fluorescence intensity (MFI), over the course of expansion for eight Treg donors. The values of LAP and GARP both increased in terms of %^+^ and MFI following activation on day 0, and these both decreased between days 3 and 11, then both increased again after restimulation on day 11. The relatively low expression of LAP and GARP at day 11 for most donors suggests that the cells had returned to a resting state and were ready to be restimulated. The fold expansion (Figure 1E) increased exponentially over time for all donors, and the mean cell diameter of the culture (Figure 1F) increased on days immediately following activation or restimulation. Figure 2 shows Raman spectra plotted for donor 5 on days 5, 7, 9, 11, 13, and 15 of expansion and depicts the characteristic patterns in the Raman spectral features over the culturing timeline. Different peaks in the Raman spectrum show dynamical values of the Raman spectral intensities over the culturing timeline, which occurs as a result of fluctuating amounts of Treg biomolecules during cell culturing. It is precisely this correlation between Raman shifts and intracellular biomolecules that is leveraged in this study to estimate levels of LAP and GARP in Tregs over the culturing timeline.

### Multisource correlation analysis shows correlation of biomarkers and spectra

**Fig. 3:**
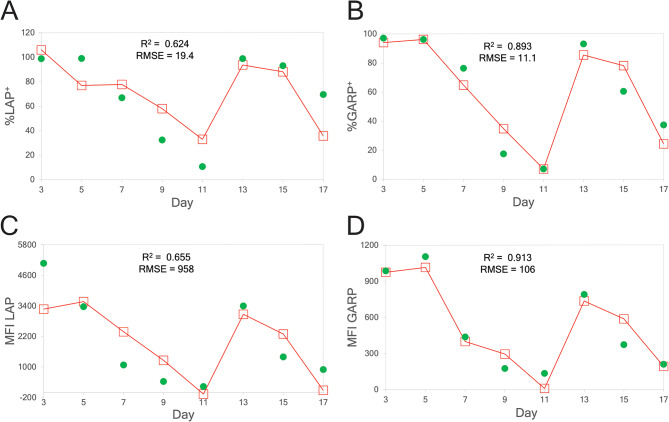
Multisource Correlation Analysis (MuSCA) estimates for the four biomarker measurements. Measured values (solid green circles) and estimates (open red squares) of four different Treg biomarkers, (**A**) %LAP^+^, (**B**) %GARP^+^, (**C**) Mean fluorescence intensity (MFI) LAP, and (**D**) MFI GARP. Measurements were made on days 3, 5, 7, ..., 17, and the solid red line is only meant to show the trend in the estimates and not meant to interpolate between data points. The estimates for each day were determined by modelling the biomarker value using a linear equation with a certain group of Raman spectral intensities. For each biomarker, MuSCA was used to manually determine the specific Raman shifts whose intensity would be used in the linear model. The day 3–17 data points for each biomarker were used in MuSCA to train the linear models.

The biomarker estimates of the preliminary Multisource Correlation Analysis (MuSCA) method^39^ are shown in Figure 3. The MuSCA methods are described in Section 1 of the Supplementary Information. Two of the biomarkers, %GARP^+^ (Figure 3B) and MFI GARP (Figure 3D), showed estimates with ${R}^{2}$ of around 0.90. The other two biomarkers in Figure 3 had estimates with relatively lower ${R}^{2}$ and higher RMSE. However, it is important to note for these results that the linear model used in the MuSCA method was trained using the same biomarker values shown in the plots in Figure 3, and therefore the corresponding estimates should be regarded as training estimates. There were limited testing estimates used in the MuSCA method, which is an impediment for our ability to analyze the robustness of the model. Nonetheless, these results showed that certain wavenumbers in the Raman spectrum were correlated with the biomarkers, motivating the subsequent work in this paper. While this preliminary model lacked the validity and rigor of the other ML models in this paper, it did reveal that this problem warranted further investigation.

### Lasso provides accurate testing scores with fewer selected features

**Fig. 4:**
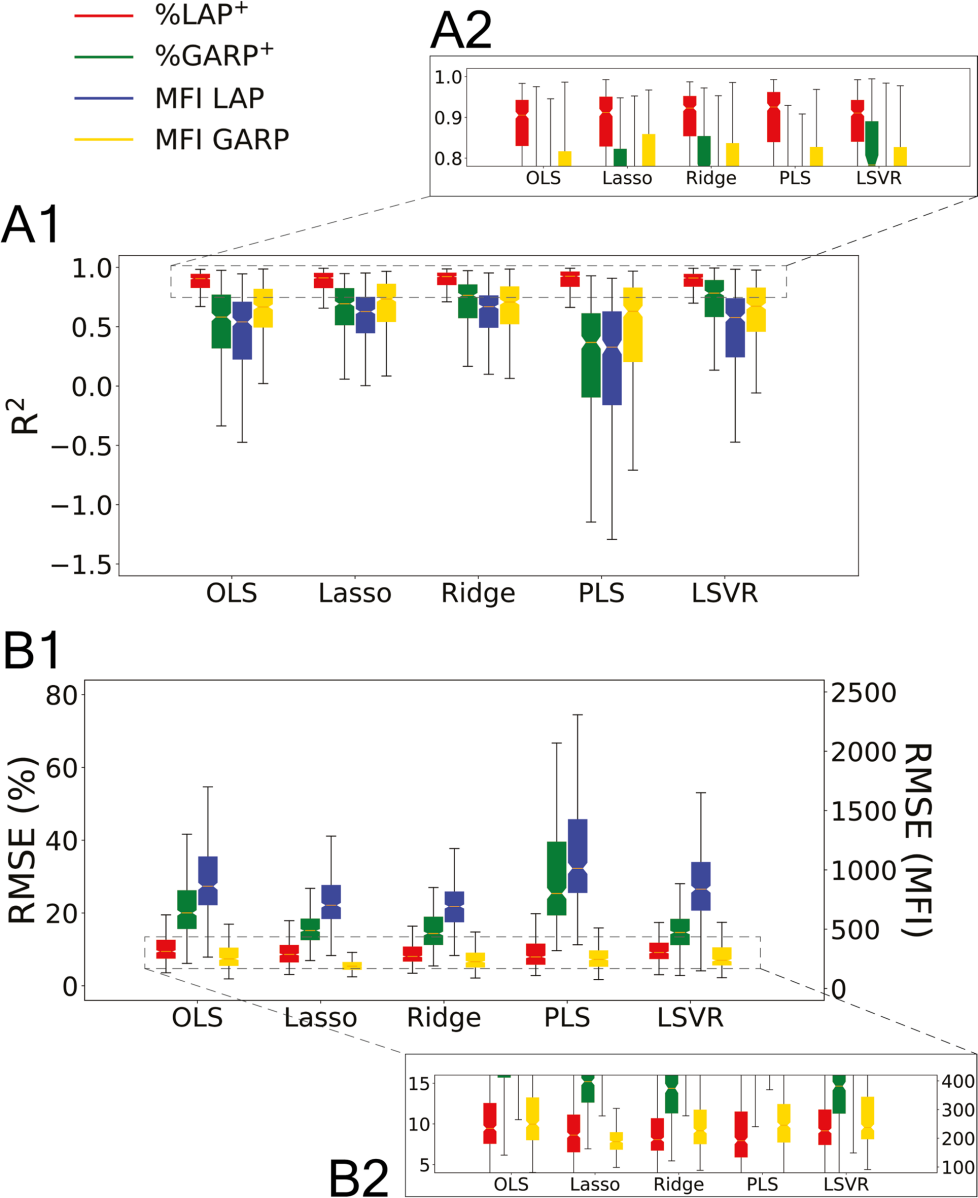
Comparison of testing scores for five machine learning models. The models Ordinary Least-Squares (OLS), Lasso, Ridge, Partial Least-Squares (PLS), and Linear Support Vector Regression (LSVR) were investigated. Boxplots for the (**A**) testing ${R}^{2}$ and (**B**) testing RMSE are shown for 50 repeated 8-fold cross validation with donors 1-8, which was performed with the optimal hyperparameters for each of the four biomarkers with each of the regularized models. For subplot B1, the RMSE of the percent positive (%) and MFI values are plotted on separate *y*-axes and are not intended to be compared. The dashed lines connecting plots A2 and B2 to plots A1 and B1, respectively, are for illustrative purposes only. The plot B2 is not an exact inset of B1, but rather it is the same plot zoomed in with different relative *y*-axis positions for the left and right *y*-axes. The plot B1 does not have the same relative *y*-axes positions as B2.

Testing accuracy scores for the five different machine learning models upon 50-repeated stratified 8-fold cross-validation are shown in Figure 4. Boxplots for the distribution of the testing ${R}^{2}$ (Figure 4A) and RMSE (Figure 4B) are plotted for each of the five models. Lasso performed at least as well as the other models in both metrics, with high median ${R}^{2}$ for all biomarkers compared to the other models. %LAP^+^ had the best testing ${R}^{2}$ scores, which was expected due to the homogeneity of the %LAP^+^ values for the eight donors in Figure 1A. Furthermore, Lasso was able to achieve this high level of accuracy with a 20- or 30-dense weight vector ${\varvec{\upbeta}}$, while OLS and Ridge typically had 963-dense weight vectors. For this reason, Lasso was chosen as the primary model for predictive analysis of novel donors because of its feature selection ability. Since Lasso was able to reduce the dimensionality of the regression from 963 to a few dozen, this served two purposes: first, it reduced the risk of overfitting by ensuring that the number of selected features used for regression was less than the number of samples; and second, it allowed for the identification of the most important biological features selected for the regression. With only 20-30 features selected in Lasso, this greatly facilitated investigating the biological assignments of the selected features to look for patterns in the weight vectors.

### Selected Lasso hyperparameter shows sparse weight vectors

**Fig. 5:**
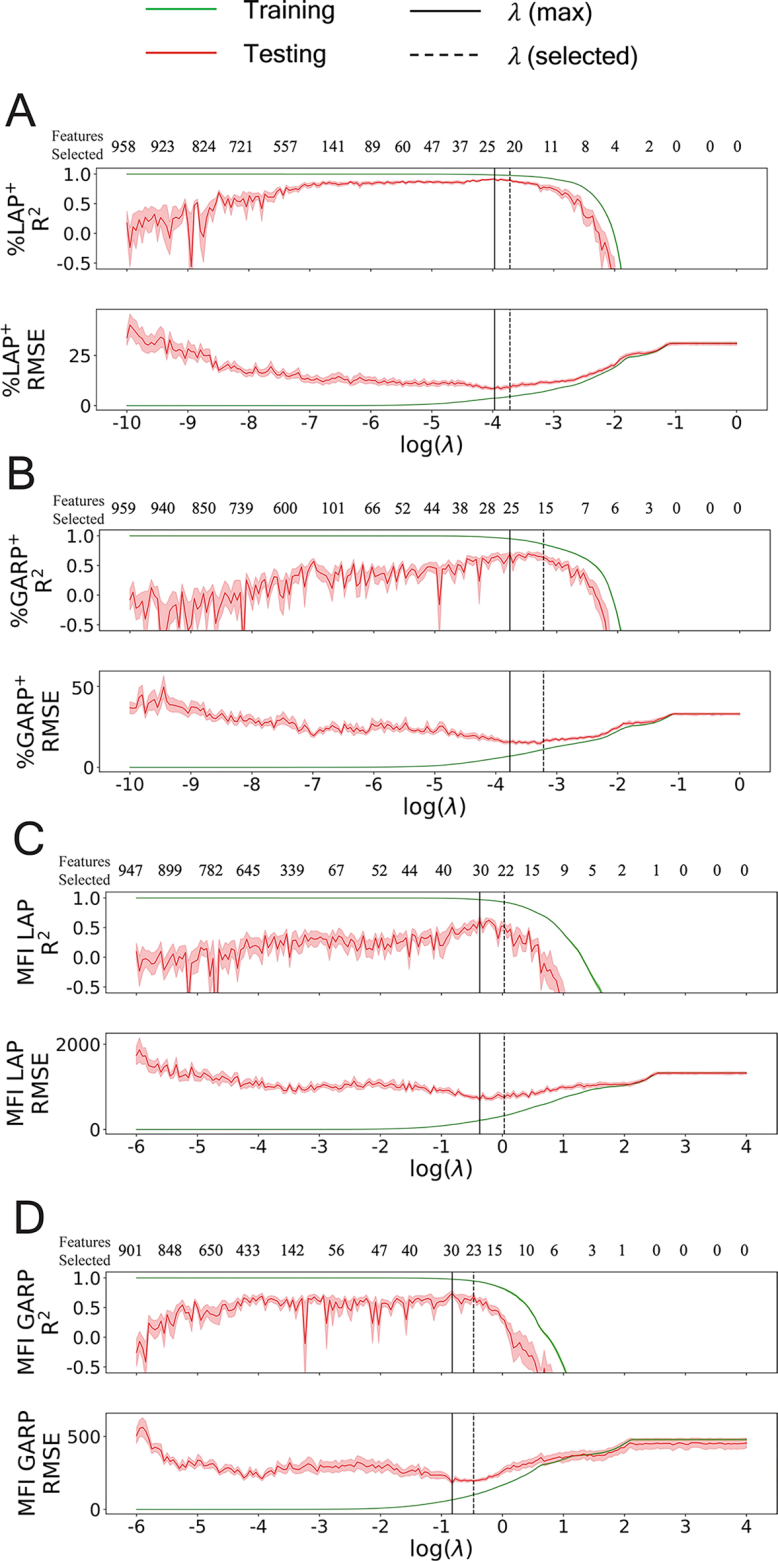
Lasso hyper-parameter tuning results for the four different biomarkers. (**A**) %LAP^+^, (**B**) %GARP^+^, (**C**) MFI LAP, and (**D**) MFI GARP. The plots show the mean training and testing ${R}^{2}$ and RMSE scores for each of the hyperparameter $\lambda$ values from all *N* epochs of 3-repeated stratified 8-fold cross validation. A total of 200 $\lambda$ values are shown in the plot. Shaded regions represent one unit of standard error. The two plots for each subfigure A–D have a shared *x*-axis. The numbers above the ${R}^{2}$ plots indicate mean number of selected features, rounded using the ceiling function. For each plot, the vertical, black solid line is the $\lambda$ value corresponding to the maximum mean testing ${R}^{2}$, and the dashed, black solid line corresponds to the selected $\lambda$, which was the largest $\lambda$ whose mean testing ${R}^{2}$ was at least as large as the lower 95% confidence interval for the maximum mean testing ${R}^{2}$.

The results of Lasso hyperparameter tuning are shown in Figure 5. The plots of ${R}^{2}$ and RMSE versus $\lambda$ showed similar trends for all four biomarkers. On the left side of the plots is the OLS limit as $\lambda \to 0$ and there was very weak regularization. The number of selected features at this end of the plots was nearly the maximum number of features, 963, for all biomarkers. As $\lambda$ increased, the number of selected features decreased and the testing ${R}^{2}$ increased while the testing RMSE decreased. For all biomarkers, the Lasso regularization appeared to increase the testing accuracy during cross-validation up to the point where about 20-30 features were selected, as indicated by the vertical, solid black lines in Figure 5. The selected $\lambda$ values, indicated by the vertical, dashed black lines in Figure 5 corresponded to a mean number of features selected of about 15-23 across the four biomarkers. On the right side of the plots in Figure 5 is the limit as $\lambda \to \infty$; in such a limit, as $\lambda$ grows boundlessly, the Lasso loss function $\mathcal{L}\left({\varvec{\upbeta}},{\beta }_{0};\lambda \right)$ will grow boundlessly and the only means of minimizing the error would be to set the 1-norm of ${\varvec{\upbeta}}$ to zero, such that the product $\lambda {\Vert {\varvec{\upbeta}}\Vert }_{1}$ remains finite. As a result, ${\varvec{\upbeta}}\to 0$ and the estimates approach a constant, ${\mathbf{Y}}^{{^{\prime}}}\to {\beta }_{0}{1}_{\text{dim}{\mathbf{Y}}^{{^{\prime}}}}$. It is for this reason that, for both the training and testing scores, the ${R}^{2}$ value becomes extremely negative and the RMSE approaches a constant as $\lambda \to \infty$.

### Lasso accurately estimates novel donor biomarkers

**Fig. 6:**
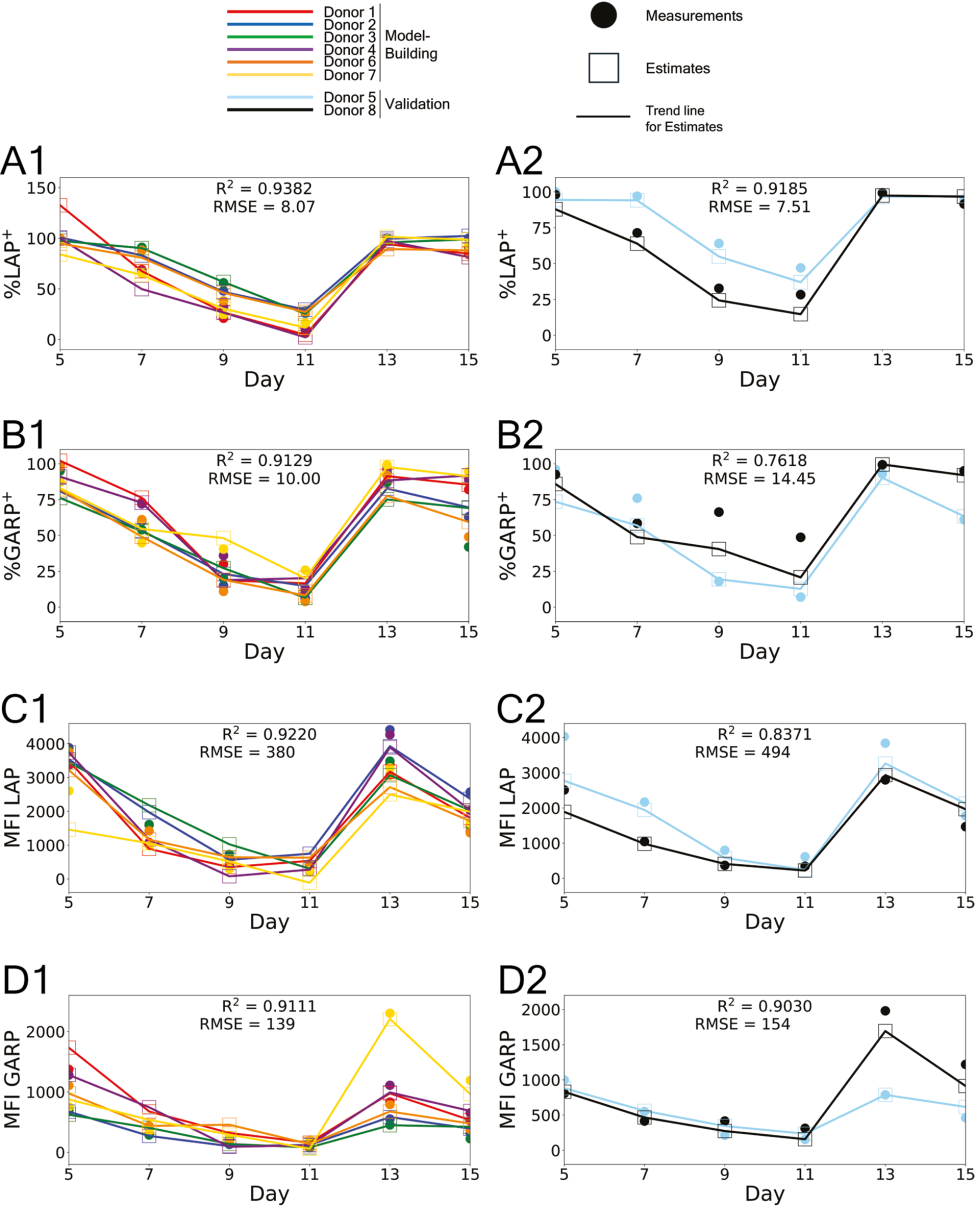
External donor validation of trained, accepted Lasso model. Estimates of the four biomarkers during days 5, 7, 9, …, 15 of culturing, with restimulation on day 11, using Lasso. Plots A1-D1 show the estimates for the model-building donors. Plots A2-D2 show the estimates for the external validation donors. The ${R}^{2}$ and RMSE values correspond to all the days and all the donors in each plot. The colors correspond to the different donors. Solid circles are biomarker measurements and open squares are the corresponding estimates. The solid line is meant to the show the trend in the estimates and is not meant to interpolate biomarker values between days.

The estimation results with the tuned Lasso model are shown in Figure 6. The trained, accepted Lasso model was used to estimate the biomarkers of the external validation donors 5 and 8. The trained weight vectors ${\varvec{\upbeta}}$ used for the estimates in Figure 6 are shown in Supplementary Tables 1-4 and described in Supplementary Section 2 of the Supplementary Information, along with the potential biological assignments of the selected features^35^. The biomarkers %LAP^+^ and MFI GARP had the most accurate estimates of the validation donors, with ${R}^{2}>0.90$. However, both %GARP^+^ and MFI LAP had validation donor estimates with ${R}^{2}>0.75$. The biomarker %GARP^+^ had the worst accuracy, which can be attributed to its high level of donor-to-donor variability; in Figure 1B, %GARP^+^ had the most variability across donors, as evident by the behavior of donors 7 and 8 between days 7 and 9 compared to donors 1-6. The trained model was unable to reliably discern this variability with such a small sample size.

In many of the weight vectors in Supplementary Tables 1-4, there were instances of a large weight corresponding to a feature in the Raman spectrum which had a low measured spectral intensity. As a result, the magnitude of the weight vector alone was insufficient to discern the importance of the selected features in the regression. As an example, in Supplementary Table 4, RNA at 811 cm^-1^ has a weight of 12461, though it only has a mean BEC of 20. Therefore, the contribution of that selected feature to the biomarker estimation is low. Other examples of features with large least-squares weights though small BECs are 485 cm^-1^ for %LAP^+^ and 1053 cm^-1^ for %GARP^+^.

## Discussion

In this work, we presented a data-driven method to analyze cultured Tregs that have many potential therapeutic applications. This work serves to illustrate the utility of Raman spectroscopy data analysis by ML methods, particularly with a relatively limited number of Treg donor sample data. The abundant and highly complex Raman spectral data from Tregs can be parsed effectively using Lasso, with which we show practical and accurate estimates of novel samples of four Treg biomarkers. The estimations in this work were obtained using simple linear regression models. Our study has laid the foundation for subsequent future work, which would involve using more advanced machine learning and deep learning methods, such as convolution neural networks, to understand the correlation between Raman spectroscopy and cell activation biomarkers. Further work would involve applying the framework in this study to other settings. This work has only studied correlations between Raman spectroscopy and flow cytometry measurements of Treg biomarkers. The ML models used in this study are robust and generalizable such that they would be able to be implemented to study correlations of other cellular biomarkers and nuclear magnetic resonance (NMR) or other spectroscopic data. As long as high-quality training data is provided, the ML models in this work would easily be trained and would estimate novel samples with high accuracy, as shown in this work.

Here, we have demonstrated the potential of Raman spectroscopy to serve as a surrogate assay for conventional bioanalytical methods which are often destructive and require sampling, and as such are not suited to on-line analyses that are desirable in large-scale cell manufacturing processes. Validating these results with on-line Raman probes integrated into cell manufacturing devices should enable improved label-free monitoring of these processes without the need for sampling. By exporting the trained model parameters for Lasso, or another ML model, we can construct a robust workflow that can estimate different biomarkers.

## Materials and methods

### Cell culture

All methods were carried out in accordance with relevant guidelines and regulations. Human research was approved by the University of British Columbia Research Ethics Board (H17-01490). Informed consent was obtained from the legal guardians of the thymus donors. Thymus-derived Tregs were isolated and expanded as previously described in MacDonald et al.^43^ Thymus donors ranged between 6 and 24 months of age and no sex discrimination was included in the analyses. In brief, thymus tissue was dissociated using the McIlwain tissue chopper (Campden Instruments Ltd., Loughborough, England) or the gentleMACS Dissociator (Miltenyi Biotec, Bergisch Gladbach, Germany), then Tregs were isolated by magnetic selection using CD25 positive selection followed by CD8 depletion (both STEMCELL Technologies, Vancouver, Canada). Isolated cells were cryopreserved in CryoStor 10 (STEMCELL Technologies) prior to expansion. Tregs were activated with Dynabeads Treg Xpander (Thermo Fisher Scientific, Waltham, MA, USA) at a 4:1 bead to cell ratio and expanded in ImmunoCult-XF T Cell Medium (STEMCELL Technologies) with 1% penicillin/streptomycin (Thermo Fisher Scientific), 1000 IU/mL recombinant human IL-2 (Proleukin, San Diego, CA, USA), and 100 ng/mL rapamycin (Sigma Aldrich, St Louis, MO, USA). Cultures were fed every 2 days starting from day 3 by adding additional media and IL-2. Additional rapamycin was added on days 3 and 5 and discontinued at day 7. Cultures were restimulated on day 11 by adding additional Dynabeads Treg Xpander at a 1:1 bead to cell ratio.

Treg samples were collected at day 0, then at 2-day intervals from day 3 of the expansion process onwards. From each sample, approximately 100-200,000 cells were used for flow cytometry and 500,000 cells saline dry-fixed for Raman spectroscopy using previously described methods^44^.

### Flow cytometry

Extracellular marker and fixable viability dye staining (Thermo Fisher Scientific) was performed in phosphate buffered saline with Brilliant Stain Buffer Plus (BD Biosciences, Franklin Lakes, NJ, USA), then cells were fixed, and intracellular staining was performed using the FOXP3/Transcription Factor Staining Buffer Set (Thermo Fisher Scientific). Data were acquired on a BD FACSymphony or BD LSRFortessa X-20. Analysis was performed using FlowJo version 10. The mean fluorescence intensity (MFI) reported was the geometric mean of the indicated population.

### Raman spectroscopy

Raman spectra were collected using an inVia Raman microspectrometer (Renishaw, Gloucestershire, UK) equipped with a 785 nm laser that generated approximately 150 mW of power at the sample. At least 60 spectra were collected per sample, with a 10 second integration time per spectrum. Each spectrum is estimated to contain information from 10-15 cells. Raman spectra were preprocessed using an automated software suite developed in-house^45^. Baseline corrected, smoothed Raman spectra were averaged and normalized to the total nucleic acid signal at approximately 782 cm^-1^ for subsequent analyses.

### Hyperparameter tuning

Optimal model hyperparameters were tuned with each biomarker for the five linear ML models. An internal script was written which followed the steps of the schematic in Supplementary Figure 1. For the $8$ donors, the script would randomly generate $8$ new blocks containing a random day 5, day 7, day 9, day 11, day 13, and day 15 spectrum. Then, the script performed 5-repeated 8-fold cross-validation by iterating through 8 epochs, in which for each epoch $i$, block $i$ would be chosen as the testing set and the remaining 7 blocks would be used as the training set. This process of 8-fold cross validation was run for 200 values of the hyperparameters within a chosen interval. For each epoch, training was performed by using the scikit-learn package in Python. The model performance metrics ${R}^{2}$ (coefficient of determination) and RMSE (root mean-square error) were determined for the training and testing biomarker values and their estimates using scikit-learn functions. Estimates were calculated using the scikit-learn functions with the trained model. For each hyperparameter value, $N=40$ cross-validation scores were obtained for all epochs over all repeats. The hyperparameter which had the highest corresponding mean testing ${R}^{2}$, denoted as ${\lambda }_{max}$ for Lasso and written similarly for the other models, was found. The standard error was calculated as $\sigma /\sqrt{N}$, where $\sigma$ is the standard deviation of the scores. The hyperparameters of Ridge, PLS, LSVR were selected to be the calculated maximum mean testing ${R}^{2}$ value. For Lasso, however, in order to decrease the number of features selected, the selected Lasso hyperparameter was the largest value satisfying $\lambda > {\lambda }_{max}$ and which had a mean testing ${R}^{2}$ larger than the lower 95% confidence interval of the ${R}^{2}$ scores for ${\lambda }_{max}$. The five machine-learning models were then evaluated with optimal hyperparameters by 50-repeated stratified 8-fold cross-validation. The distributions of testing ${R}^{2}$ and RMSE were compared for model selection.

### Model training

The validation set was constructed by selecting one of donors 1-6 and one of donors 7-8, both at random. The remaining donors then comprised the model-building set (Supplementary Figure 2). The model training was performed in Python using scikit-learn functions. The model-building set was randomly split into training and testing sets, which were 70% and 30% of the original set, respectively, and the Lasso model was then trained with the training set, after which the estimates of the training and testing $\mathbf{Y}$ vectors were obtained. For analysis of the training and testing estimates compared to the true biomarker values, the metrics ${R}^{2}$ and RMSE were determined. The model was only accepted if the ${R}^{2}$ between the measured testing biomarker values of the model-building set, ${\mathbf{Y}}_{test}$, and the corresponding estimated testing set biomarker values, ${\mathbf{Y}}'_{test}$, was ${R}^{2}>0.90$; otherwise, the random splitting and training were repeated. This artificially induced model-selection step was performed to ameliorate the issue of low sample sizes which exist in works with clinical samples, including this work. The purpose of this model-selection step was to show that there exist some model parameters which can be obtained from the model-building set donors, which are able to be exported and used for robust estimations of biomarker values in novel settings. Specifically, this selection was done to find the best parameters given the small sample size. The accepted trained model was then used to estimate the biomarker values of the validation set. Thus, we implemented the model-validation step after model-selection to ensure the accuracy and robustness of the final model despite this artificial selectivity.

## Supplementary Information

Below is the link to the electronic supplementary material.

Supplementary Material 1

## Data Availability

The data and code used to generate the figures in this work is available on a public repository at https://github.com/daisubc.
