# Supplementary material for: Label-free estimation of regulatory T cell activation markers using Raman spectroscopy with machine learning
Source: Sci Rep. 2025 Nov 4;15:38519. doi: 10.1038/s41598-025-16002-8 (PMC12586467; doi:10.1038/s41598-025-16002-8)
Supplement: Supplementary file 1 — Supplementary Material 1 [file 41598_2025_16002_MOESM1_ESM.pdf]

Supplementary Information for
Label-Free Estimation of Regulatory T Cell Activation Markers Using Raman Spectroscopy with Machine Learning

Aria Azari-Pour, Ali Chamkalani, Shreyas Rangan, Katherine N. MacDonald,

Miles Huynh, Megan K. Levings, H. Georg Schulze, James M. Piret, Bhushan Gopaluni

1 Multisource Correlation Analysis

The results of the Multisource Correlation Analysis (MuSCA) were obtained according to Schulze et al.[1] Using the normalized spectra, MuSCA extends 2D correlation spectroscopy to include biochemical data, such as %LAP$^+$ or another biomarker, which ultimately outputs the two-dimensional correlation value (TDCOR) for each Raman shift in terms of how it relates to biochemical data. A TDCOR of $+1$ refers to perfectly correlated, 0 refers to no correlation, and -1 refers to perfectly anticorrelated. TDCOR values larger than approximately $0.55 - 0.70$ were considered, which was chosen to ensure approximately $3 - 4$ clusters of Raman shifts were correlated with parameters. To process and understand the TDCOR values and the Raman shifts associated with biochemical data, some further filtering was performed where the average intensity below the limit of detection (three times the spectral intensity) was filtered out; the remaining shifts with the highest TDCOR values were sorted, and then clusters of at least three Raman shifts less than 6 cm^{-1} apart were kept. This removed near-zero, high error shifts and avoided isolated Raman shifts, so that any assignments were more likely to be related to a shift assignment than random overfitting. The model was developed using the available data for all days at the start to verify that it worked (all training, no testing) and eventually using days $3 - 11$ for training and days $13 - 19$ for testing.

2 Biological Assignments of Selected Features Reveal Unique Patterns

Following training and acceptance of the Lasso models for each of the biomarkers, the different weight vectors were analyzed for their profiles of selected features and biological assignments. The results are summarized in Supplementary Tables **1** – **4**. We observed different trends for weights of selected features compared to their mean BEC. There were selected features with large weights but a small mean BEC; such a result occurs when a selected feature has a Raman spectral intensity that is very small over the culturing timeline. An example of this occurred for MFI GARP (Supplementary Table **4**) with the selected Raman shift at 811 cm^{-1}. The corresponding RNA peak in the Raman spectra for this Raman shift is known to have a very low intensity, so even though the weight for this feature was $\beta_{811\ \mathrm{cm}^{-1}} = 12461$, the mean BEC in Supplementary Table **4** was only 20. We therefore concluded that the magnitudes of the weight vector components were insufficient to completely determine the extent to which selected features impacted the prediction of new samples, and our analysis of BEC served as a better metric for ranking selected features in terms of their importance for predictions. Implicit in this conclusion is the assumption that the relative magnitudes of spectral intensities remained constant across donors and days, to within some allowed variation during the culturing timeline. From our results, this assumption is valid for our work with Tregs but may not be the case for other types of T cells.

Also of interest are the specific Raman features selected for the different biomarkers. Though these are different biomarkers and were established via different methods, one might have expected more overlap between Raman features identified for a given biomarker. For example, for the LAP results, essentially the same activation-related internal cellular processes must be involved in its surface expression even though subsequently assessed either as percent

positive or mean fluorescence intensity. Those aspects of these LAP related cellular processes recorded with Raman spectroscopy must then be the same for %LAP$^+$ and MFI LAP. However, only adenine (~727 cm^{-1}), phenylalanine (~1003 cm^{-1}), the DNA backbone (~1100 cm^{-1}) and possibly cholesterol seem to be in common (Supplementary Tables **1** and **3**). Such detailed analyses and interpretations of selected Raman features, their biological significance and their specific relationships to the respective biomarkers and their measurement methods will need to be addressed in future work.

References

1. Schulze, H. G. et al. Augmented Two-Dimensional Correlation Spectroscopy for the Joint Analysis of Correlated Changes in Spectroscopic and Disparate Sources. *Appl. Spectroscopy* **75**, 520–530 (2021). https://doi.org/10.1177/0003702820979331.

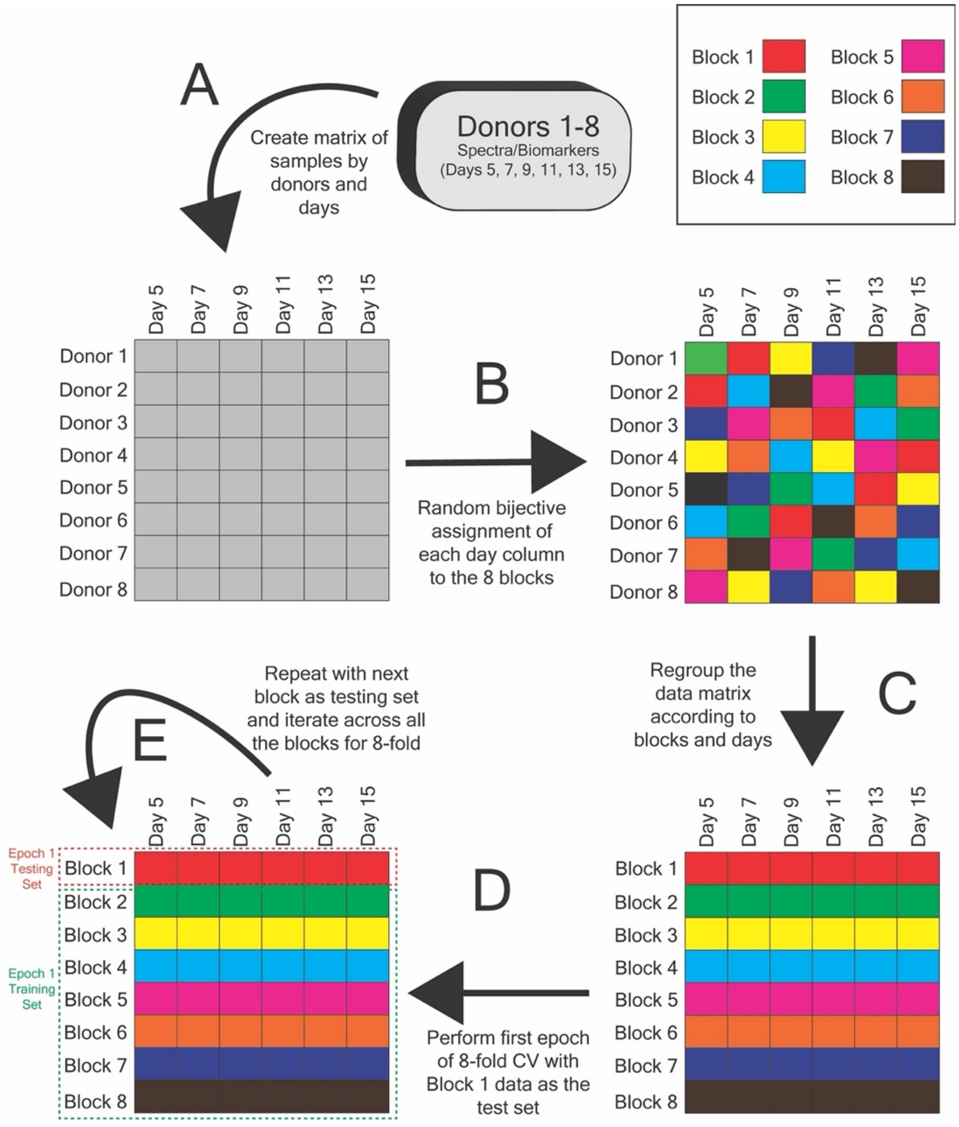

Supplementary Figure 1. Schematic of stratified k-fold cross-validation method used for hyperparameter tuning of the machine learning models. (A) The samples (spectra and biomarkers) depend on the sample donor and sampling day. The 48 samples can be arranged in a matrix. (B) There are 8 experimental groups (*blocks*), one for each donor, that are randomly assigned to the 8 donors in each day column in the matrix as a bijection. (C) New rows are made that correspond to the experimental blocks, a process that is referred to as re-donorization, and which completes stratification. Cross-validation (CV) is performed by running $k = 8$ epochs in which block i is the testing set of the ith epoch and the other 7 blocks are the training set. Both root-mean-square error (RMSE) and the coefficient of determination (R^2) are calculated as metrics for each epoch, leading to 8 scores for each metric. Repeated k-fold CV was performed by repeating the entire process from (A) – (E) *n* times for a total of 8*n* scores for each metric.

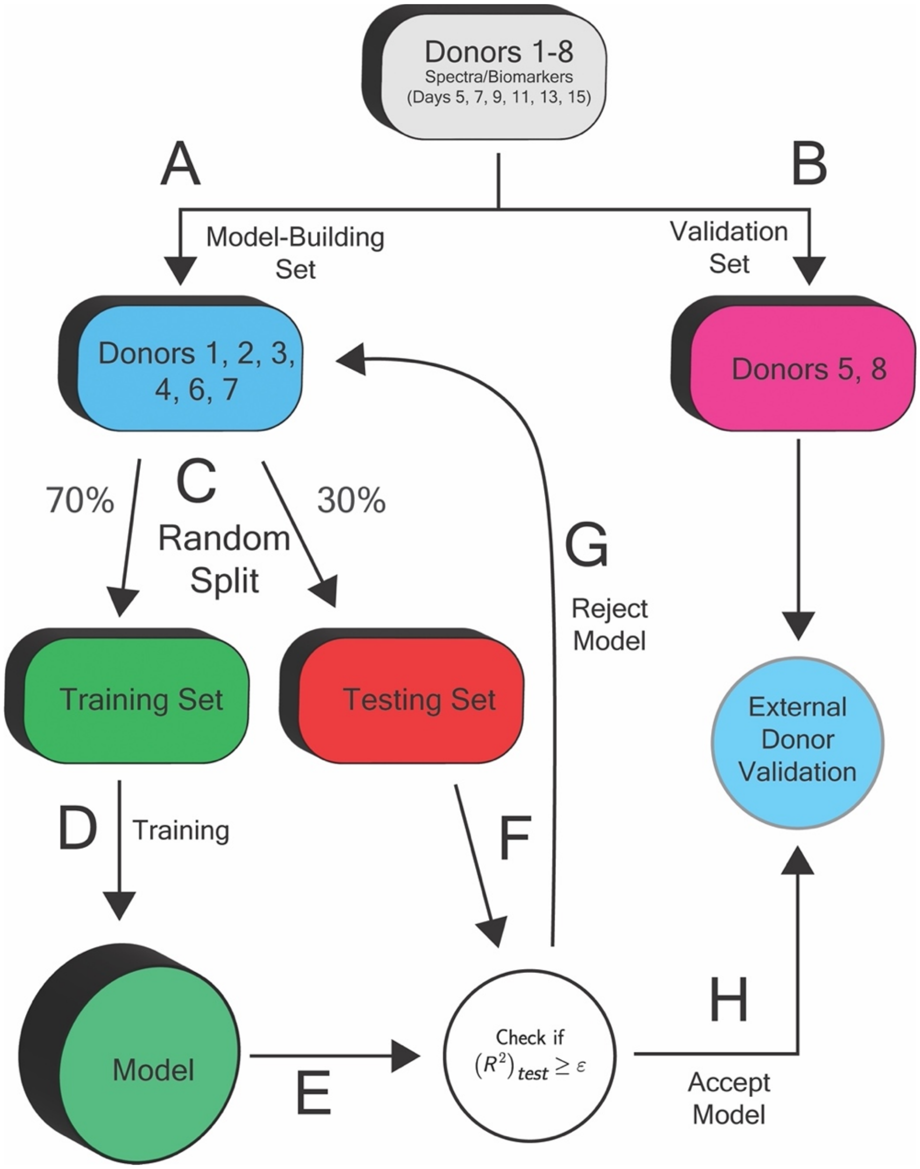

Supplementary Figure 2. Schematic for machine learning model-building and validation. Initially, the 8 donors were randomly separated into two sets, (A) the model-building set and (B) the validation set. (C) The model-building set was randomly split into training and testing sets, with sizes of 70% and 30% of the model-building set, respectively. (D) The parameters of the model are found using the training set, and then (E, F) the model is used to estimate the biomarkers of the testing set from their corresponding Raman spectra. (G) If the testing R^2 is less than a pre-determined threshold, ε, then the model is rejected, and a different random splitting of the model-building set is performed. (H) Otherwise, the model is accepted and used to estimate the biomarkers of external donors.

%LAP⁺				
**Raman Shift (cm^{-1})**	**Assignment**	**Weight**	**Mean BEC**	**SD BEC**
422	Cholesterol	−66.5	−13.0	3.83
485	Glycogen	164.0	12.6	6.36
497	DNA	−55.8	−19.5	2.94
501	DNA	−11.8	−3.50	0.649
502	DNA	−87.3	−22.8	4.63
728	Adenine	−9.1	−6.11	0.660
729	Adenine	−27.6	−15.5	1.85
829	Tyrosine, O–P–O stretch (DNA)	−86.6	−34.9	4.43
999	Phenylalanine	−26.3	−30.0	1.71
1000	Phenylalanine	−21.6	−40.7	1.96
1005	Phenylalanine	−6.0	−20.4	1.54
1007	Phenylalanine	−7.4	−12.6	1.24
1030	Phenylalanine	89.4	49.9	4.25
1032	Phenylalanine, $\delta(\mathrm{C-H})$	62.3	38.8	3.27
1047	Proline	−27.8	−0.944	0.707
1049	Proline	−9.96	−0.274	0.232
1102	Phenylalanine, O–P–O stretch (DNA)	−162.9	−113.6	10.4
1127	Lipids, Proteins (C-N stretch)	40.6	31.0	4.11
1128	Lipids, Proteins (C-N stretch)	16.2	11.7	1.81
1129	Lipids, Proteins (C-N stretch)	17.4	11.2	1.98
1307	Lipids	50.9	42.7	4.56
1308	Lipids	14.8	12.7	1.18

Supplementary Table 1. **Raman shifts and biological assignments associated with %LAP$^+$ for Figure 6 of the main text.** The non-zero weights for each Raman shift in the linear regression equation defined in the main text are shown for the biomarker %LAP$^+$. The least-squares weights for each biomarker have dimensions [biomarker][Raman shift]$^{-1}$ and the BEC (Biomarker Estimation Contribution) has dimensions [biomarker].

%GARP$^+$				
**Raman Shift (cm^{-1})**	**Assignment**	**Weight**	**Mean BEC**	**SD BEC**
496	DNA	−23.8	−8.23	1.22
497	DNA	−67.9	−23.8	3.57
729	Adenine	−258.8	−145.7	17.3
844	Unclear	−237.7	−26.1	7.33
1031	Phenylalanine	61.1	34.1	2.91
1033	Phenylalanine	69.1	41.5	3.79
1053	Proteins (C–O stretch, C–N stretch)	−54.8	−0.445	0.551
1100	O–P–O stretch (DNA)	−25.2	−16.2	1.41
1316	Guanine	21.6	18.9	0.776
1340	Adenine, Guanine	−103.3	−113.8	16.8
1437	Proteins, Lipids (CH_2 scissoring)	−29.9	−44.9	6.60
1455	Proteins, Lipids (CH_2 stretching/CH_3 asymmetric deformation)	48.3	129.6	11.9
1456	Proteins, Lipids (CH_2 stretching/CH_3 asymmetric deformation)	2.1	5.38	0.486
1477	Guanine, Adenine	48.6	9.34	1.35

Supplementary Table 2. Raman shifts and biological assignments associated with %GARP$^+$ for Figure 6 of the main text. The non-zero weights for each Raman shift in the linear regression equation defined in the main text are shown for the biomarker %GARP$^+$. The least-squares weights for each biomarker have dimensions [biomarker][Raman shift]$^{-1}$ and the BEC (Biomarker Estimation Contribution) has dimensions [biomarker].

MFI LAP				
Raman Shift (cm^{-1})	Assignment	Weight	Mean BEC	SD BEC
418	Cholesterol	−3065	−649	150
543	Cholesterol	−3455	−464	134
557	No Assignment	−4589	−371	50
597	Phosphatidylinositol	32896	2959	597
644	Tyrosine	−8849	−5332	543
646	Tyrosine	−1992	−645	89
714	Phosphatidylcholine	−2699	−685	52
727	Adenine	−4223	−3183	303
844	Unclear	−2745	−301	85
854	Tyrosine	1153	841	78
855	Tyrosine	2002	1314	126
1004	Phenylalanine	−49	−186	12
1005	Phenylalanine	−1505	−5070	385
1043	Proline	−1321	−103	40
1094	DNA	−1222	−538	47
1101	DNA (O–P–O backbone stretch)	−192	−130	11
1172	Tyrosine	−154	−31	5
1238	Amide III	−264	−113	6
1256	Amide III, Lipids, DNA	1567	469	121
1329	Nucleic acids (CH_3CH_2 wagging modes), Phospholipids	2047	1919	196
1435	Proteins, Lipids (CH_2 scissoring)	2316	2137	355
1472	Lipids	12695	6205	519

Supplementary Table 3. **Raman shifts and biological assignments associated with MFI LAP Figure 6 of the main text.** The non-zero weights for each Raman shift in the linear regression equation defined in the main text are shown for the biomarker MFI LAP. The least-squares weights for each biomarker have dimensions [biomarker][Raman shift]$^{-1}$ and the BEC (Biomarker Estimation Contribution) has dimensions [biomarker].

MFI GARP				
**Raman Shift (cm^{-1})**	**Assignment**	**Weight**	**Mean BEC**	**SD BEC**
492	DNA	−340	−99	15
493	DNA	−603	−189	28
644	Tyrosine	−3709	−2235	228
668	Cystine, Thymine, Guanine	1536	355	35
724	Adenine	−514	−399	28
727	Adenine	−261	−196	19
811	RNA	12461	20	20
876	Hydroxyproline, Tryptophan	358	42	8
1002	Phenylalanine	−395	−1433	71
1008	Phenylalanine	682	752	74
1064	Lipids (C–C stretch)	−3026	−186	98
1094	DNA, Proteins (C – N stretch)	−1254	−553	49
1111	DNA	3005	1291	109
1132	Proteins, Lipids (C – C stretch)	−691	−202	52
1229	Amide III	811	299	23
1338	Proteins, Lipids (CH_2/CH_3 wagging, twisting, or bending modes), Adenine, Guanine	−1777	−2011	275
1439	Lipids	−79	−159	21
1456	Deoxyribose, Proteins, Lipids (CH_2 stretching/CH_3 asymmetric deformation)	1616	4134	374

Supplementary Table 4. **Raman shifts and biological assignments associated with MFI GARP for Figure 6 of the main text.** The non-zero weights for each Raman shift in the linear regression equation defined in the main text are shown for the biomarker MFI GARP. The least-squares weights for each biomarker have dimensions [biomarker][Raman shift]$^{-1}$ and the BEC (Biomarker Estimation Contribution) has dimensions [biomarker].
